# Evidence that *PICALM* affects age at onset of Alzheimer's dementia in Down syndrome

**DOI:** 10.1016/j.neurobiolaging.2013.03.018

**Published:** 2013-10

**Authors:** Emma L. Jones, Kin Mok, Marisa Hanney, Denise Harold, Rebecca Sims, Julie Williams, Clive Ballard

**Affiliations:** a Wolfson Centre for Age-Related Diseases, King’s College London, London, UK; b Reta Lila Weston Laboratories and Departments of Molecular Neuroscience, UCL Institute of Neurology, Queen Square, London, UK; c MRC Centre for Neuropsychiatric Genetics and Genomics, Cardiff University, Henry Wellcome Building for Biomedical Research, Heath Park, Cardiff, UK

**Keywords:** Genome-wide association study, Down syndrome, Alzheimer’s disease, *PICALM*, *APOE*

## Abstract

It is known that individuals with Down syndrome develop Alzheimer’s disease with an early age at onset, although associated genetic risk factors have not been widely studied. We tested whether genes that increase the risk of late-onset Alzheimer’s disease influence the age at onset in Down syndrome using genome-wide association data for age at onset of dementia in a small sample of individuals (N = 67) with Down syndrome. We tested for association with loci previously associated with Alzheimer’s disease risk and, despite the small size of the study, we detected associations with age at onset of Alzheimer’s disease in Down syndrome with *PICALM* (β = 3.31, *p* = 0.011) and the *APOE* loci (β = 3.58, *p* = 0.014). As dementia in people with Down syndrome is relatively understudied, we make all of these data publicly available to encourage further analyses of the problem of Alzheimer’s disease in Down syndrome.

## 1 Introduction

Down syndrome (DS), also referred to as trisomy 21, is caused by an extra copy of chromosome 21. Occurring in approximately 1 in 800 live births, it is the most common chromosomal abnormality resulting in learning difficulties.

The prevalence of Alzheimer's disease (AD) in people with DS increases significantly with age, and dementia in people with DS is one of the most common forms of dementia among individuals under the age of 50 years. Despite the occurrence of AD pathology, including amyloid plaques and neurofibrillary tangles, in the brains of individuals with DS as young as 40 years (Wisniewski et al., 1994), the age at onset (AAO) of AD can vary widely. The mean AAO of dementia is 50 to 55 years (Prasher and Krishnan, 1993), with a range from 38 to 70 years.

The early-onset AD that occurs in people with DS is thought to be largely due to the triplication of *APP*, which is located on chromosome 21. Indeed, *APP* duplications have been shown to cause early-onset AD in the disomic population (McNaughton et al., 2012; Rovelet-Lecrux et al., 2006; Sleegers et al., 2006), although these are not always fully penetrant (Hooli et al., 2012).

As dementia in the DS population can develop over a range of ages, it is likely that, as with sporadic AD in the general population, genetic factors play a major role. Although mutations in a small number of genes associated with the production of amyloid, such as *APP*, *PSEN1,* and *PSEN2*, have been found to cause early-onset AD in the general population, the majority of AD cases are sporadic and are likely to have a complex genetic etiology. *APOE* is a key genetic risk factor for late-onset AD in the general population (Corder et al., 1993), and large genome-wide association studies (GWAS) carried out in recent years have identified further risk genes: *CLU*, *PICALM*, *BIN1*, *CR1*, *ABCA7*, *CD2AP*, *EPHA1*, *CD33,* and the *MS4* locus (Harold et al., 2009; Hollingworth et al., 2011; Naj et al., 2011; Seshadri et al., 2010). Although we and others have shown *APOE* to affect AD risk in DS individuals (Deb et al., 2000; Prasher et al., 2008; Royston et al., 1996), this result has not been replicated in all studies (Margallo-Lana et al., 2004; van Gool et al., 1995).

A study by Patel et al. (2011) examined SNPs in 28 genes associated with AD risk, selected from literature published up until March 2008. Five genes including *APOE* were associated with AD risk.

No therapies have specifically been developed for the treatment of AD in DS, and, as the life expectancy of people with DS is increasing, it is becoming more important to understand the factors affecting age-related diseases in this population (Coppus et al., 2006; Holland et al., 2000).

Given the almost universal presence of early-onset AD pathology in people with DS, factors affecting AAO, severity, and disease progression need closer analysis. This may also provide information relevant to those at risk of developing AD in the general population. In this study, we examined the impact of SNPs that have recently been associated with AD risk in the general population, in 2 large meta-analyses (Hollingworth et al., 2011; Naj et al., 2011), upon AAO of AD in people with DS.

## 2 Methods

### 2.1 Samples

A total of 158 DNA samples were prepared from blood or brain from individuals with DS using a commercially available kit (DNeasy Blood and Tissue kit, Qiagen). Ninety-four blood samples were collected from two clinical trials (MEADOWS (Hanney et al., 2012), DOWNSLIT [http://public.ukcrn.org.uk/search/StudyDetail.aspx?StudyID=5927]), and 64 brain samples from the Newcastle and London Neurodegenerative Disease brain banks, and Thomas Willis Oxford Brain Collection, as part of the Brains for Dementia Research initiative, the Medical Research Council Alzheimer Brain & Tissue Bank in Edinburgh, the NICHD Brain and Tissue Bank for Developmental Disorders, University of Maryland (NICHD Contract No. N01-HD-4-3368 and NO1-HD-4-3383), the Alzheimer’s Disease Research Center, Washington University in St. Louis, and the Netherlands Brain Bank. All samples were anonymous to the researchers carrying out the genotyping and genotyping analysis. Samples were requested and collected with permission from North West, and Newcastle and North Tyneside Research Ethics Committees. Samples were collected with informed consent using processes approved for each brain bank or by the Research Ethics Committees.

The MEADOWS trial examined the use of memantine as a treatment for cognitive decline in DS over the course of 52 weeks. Similarly, the DOWNSLIT trial used lithium to improve cognitive function in DS over the course of 8 weeks. The use of memantine in the MEADOWS trial did not alter the risk of developing dementia or AAO (χ^2^ = 0.031, *p* = 0.861 and *t* = 0.291, *p* = 0.745 respectively). No individuals in the DOWNSLIT trial had a diagnosis of or developed dementia. Cohort demographics are shown in Table 1. There were no differences in gender frequencies between the blood and brain samples cohorts (χ^2^ = 0.013, *p* = 0.908). Individuals in the autopsy cohort were older than those in the clinical cohort (Mann–Whitney *U* test, *p* = 0.008).

**Table 1:** Age and gender of clinical and autopsy cohorts for all samples, and for the 67 samples used in the age at onset (AAO) analysis

Cohort	Age, y (mean ± SD)	Gender (% male)
Clinical	46.61 (10.63)	55.3
Autopsy	49.03 (16.95)	56.3
Clinical (AAO)	54.18 (5.45)	52.9
Autopsy (AAO)	55.72 (7.97)	52.0

For samples from the clinical trials, AAO of dementia was recorded by the trial psychiatrist (M.H.) and the youngest AAO was 34 years. The mean duration of dementia before death in people with DS is approximately 5 years (Prasher and Krishnan, 1993; Prasher et al., 2008). For the autopsy cohort, diagnosis of dementia and AAO had not been routinely collected. Therefore we defined AAO as age of death minus 5 years for all individuals over the age of 34. This, of course, assumes that all individuals over this age had dementia.

### 2.2 Genotyping and quality control

DNA was genotyped in the UCL Genomics Centre using HumanOmniExpress-12v1_H beadchips. The data were assembled in Genome Studio (Illumina, San Diego, CA). A total of 158 samples were genotyped. After the initial quality control check, 129 samples (72 male and 57 female) and 642,251 SNPs remained.

SNPs were first curated in Genome Studio. Those SNPs in Chr 1 to 22 that failed the call frequency parameter (defined as <0.98) were analyzed by re-cluster in Genome Studio. Within this group, only those SNPS that passed quality parameters were kept (namely Het excess between 0.1 and −0.1 and cluster separation >0.3). All others were discarded (zeroed in Genome Studio). Samples with call rates <0.975 after this process were excluded (9 samples and 29,537 SNPs were excluded in this step).

Samples were curated in PLINK (Purcell et al., 2007) for gender check and relatedness (defined as PiHat >0.125), and SNPs were checked for minor allele frequency (≥0.01), haplotype missingness (*p* < 10e-4), and Hardy–Weinburg Equilibrium (*p* < 10e-6). Ten samples were excluded (6 were duplicates of another sample in the study). 58,737 SNPs were excluded at this stage, of which 58,405 were due to MAF <0.01. The outcome of this process resulted in 139 samples (61 female and 78 male) and 642,251 SNPs for further analysis.

Samples were merged with Hapmap data. MultiDimensional Scaling on the combined samples was used for assessing population outliers. Those samples that were 4 standard deviations away from the mean of CEU/TSI combined data were excluded. Ten samples were found to be nonwhite or mixed. At this stage, only 129 samples (72 male and 57 female) and 642,251 SNPs remained.

As dementia develops at a mean age of 50 to 55 years, and in our clinical sample series, the earliest age of dementia onset was 34 years, we excluded all cases with age <34 at their last cognitive assessment or autopsy. This resulted in 120 valid samples with 70 having dementia (clinically defined, or belonging to the autopsy cohort). Among the 70 dementia cases, 3 had no AAO in the record. The analysis was based on 67 cases (Table 1). The youngest age in the autopsy series was 39, and therefore AAO in this case was 34 as defined above. There were no differences in gender frequencies between the clinical and autopsy samples used at this stage (χ^2^ = 0.005, *p* = 0.946) or age (Mann–Whitney *U* test, *p* = 0.419).

The MEADOWS trial showed that memantine was not an effective treatment for cognitive decline in DS (Hanney et al., 2012). There were no AAO differences between those taking placebo and memantine in the 17 samples used for the AAO analysis (*t* = −0.621, *p* = 0.545). No individuals from the DOWNSLIT trial developed dementia, and so were excluded from this further analysis.

Analysis was carried out in PLINK using a linear regression with AAO and the SNPs in an additive model. The SNPs were selected from previous GWAS meta-analyses (Hollingworth et al., 2011; Naj et al., 2011) and had reached statistical significance or possible significance (Supplementary Table 8 (Hollingworth et al., 2011), Tables 1 and 2, and Supplementary Table 5 (Naj et al., 2011)). Of the 531 SNPs found in these 2 papers, only 157 SNPs were also successfully genotyped by HumanOmniExpress-12v1_H in this study (most were absent in this array). A detailed list can be found in Table S1. Among these, 35 SNPs from 15 genes were around the *APOE* region.

Component 1 and component 2 in PLINK MultiDimensional Scaling were used as covariates, in addition to gender and the classification of the sample as clinical or autopsy. A nominal *p* value of 0.05 was used as a cut-off for further analysis.

R was used for graph plotting (R Development Core Team, 2008).

### 2.3 Risk score analysis

We generated a composite score of AD risk allele loading based on the SNPs reported by Bettens et al. (2013). We correlated the age of onset in our DS cases with the composite score derived from AD studies. In addition, we compared AAO between the quintiles with highest and lowest score. For details, please refer to the Supplementary Method (MethodS1).

## 3 Results

**Fig. 1:**
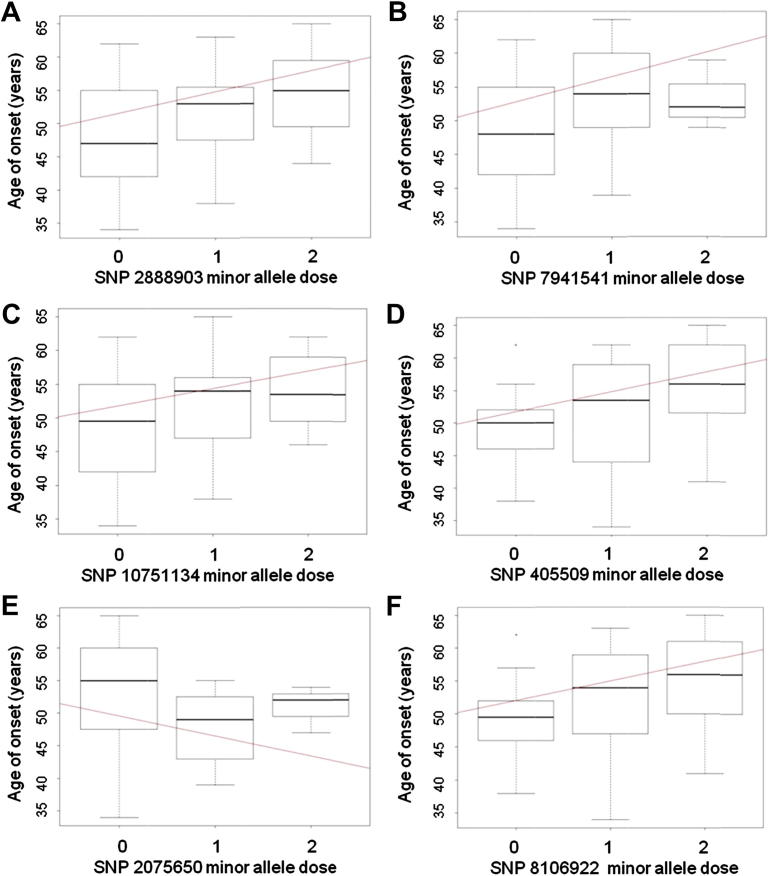
Median age of onset of dementia according to genotype. 0 = homozygous major allele, 1 = heterozygote, 2 = homozygous minor allele. A = rs2888903 (T/G). B = rs7941541 (A/G). C = rs10751134 (A/G). D = rs405509 (C/T). E = rs2075650 (A/G). F = rs8106922 (A/G).

We found a significant association between AAO and variation at the chromosome 11 *PICALM* region. Three SNPS, rs2888903 (β = 3.31, *p* = 0.011), rs7941541 (β = 3.92, *p* = 0.016), and rs10751134 (β = 2.78, *p* = 0.040) were associated (Fig. 1A–C). All of these *PICALM* SNPs show a decreased AAO in individuals who are homozygous for the major allele.

There is also a significant association between AAO and *APOE,* replicating associations found with AD risk in previous studies. The median AAO in patients homozygous for the minor allele in rs405509, a SNP linked with *APOE*, was 5 years older than those homozygous for the major allele (β = 3.58, *p* = 0.014), (Fig. 1D). Two SNPS in *TOMM40*, which is immediately 5′ to *APOE*, were also associated with AAO; rs2075650 (β = −3.4, *p* = 0.036, Fig. 1E) and rs8106922 (β = 3.32, *p* = 0.031, Fig. 1F). These *PICALM*, *APOE* and *TOMM40* SNPs show the same direction of effect as in studies in the general population: that is, an increased risk of AD in the general population equates with an earlier AAO in the DS population in this study.

The only SNP in our study that showed a significant effect in the direction opposite to that found in the general population was rs3865444 in CD33; but it is not protective, as was found in the AD GWAS studies (Table S3). The other genes previously found to have been associated with AD risk did not have significant associations with AAO of AD (Table S3).

**Figure S1:**
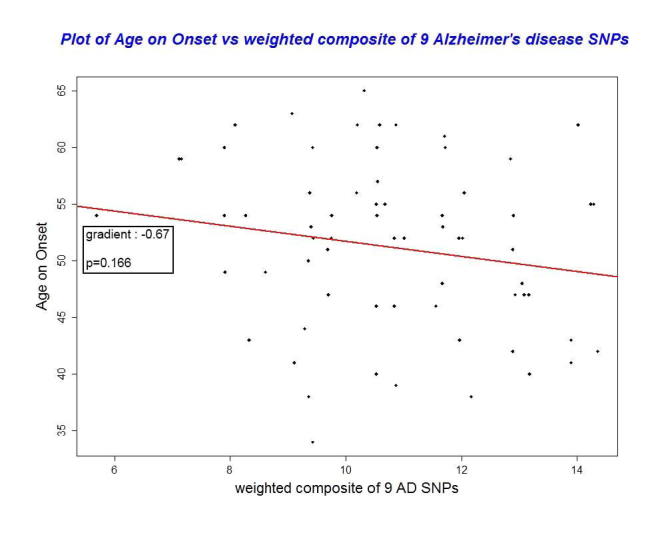


With regard to risk score analysis, when comparing the upper and lower quintiles, AAO changed from 48.1 (±SD 6.3) to 52.9 (±SD 6.9) (Table S4) (Mann–Whitney *U* test, *p* = 0.084). There is a trend toward an association between AAO and allele composite score (*p* = 0.166) (Figure S1). For every increase of 1 in the composite score, AAO decreased by 0.67 year. All tests for the composite score were insignificant if APOE SNP rs405509 is excluded.

## 4 Discussion

The major alleles of the 3 SNPS in *PICALM*, rs2888903, rs7941541, and rs10751134, are associated with an increased risk of AD in the general population and an earlier AAO in the DS population. A further SNP in *PICALM*, rs561655, whose association with AD was the most significant in the study by Naj et al. (2011) was not significantly associated with AAO of AD in our cohort (data not shown), but had the same protective result as that found by Naj et al. (2011). The 3 *PICALM* SNPs noted in this study are in linkage disequilibrium (LD). A further *PICALM* SNP that has been associated with AD in the general population, rs3851179, is in LD with both rs7941541 and rs10751134. As with rs561655, we do not find this SNP to be significantly associated in our study, but its effect is protective, as found in the general population.

Similarly, the results for the *APOE* SNP rs405509 and *TOMM40* SNP rs8106922 are in accordance with the study by Naj et al. (2011), with the minor allele having a protective effect upon the onset of dementia. The minor allele of rs2075650 in *TOMM40* is associated with increased risk of AD (Naj et al., 2011) and an earlier AAO in our current study, although this is most likely due to LD with the *APOE* locus.

With regard to the composite risk score analysis, the level of significance is likely limited by our sample size; but this analysis has shown that using a composite score may help in predicting the risk and should be one of the directions in future studies.

The score is likely dominated by APOE, given that the other SNPs have a much lower OR in the AD meta-analyses.

This GWAS was powered to detect only the risk associated with *APOE*, and is too underpowered to detect the many more subtle changes associated with AD risk in the general population. Although *APOE* has previously been associated with the risk of developing AD in individuals with DS, *PICALM* has not. This study therefore highlights the value of carrying out GWAS in the DS population. Even with a small sample size, we are examining a population with a more homogeneous AD risk than that of the general population, and by using the results of previous GWAS to focus our study, we can detect genetic risk factors that have not yet been associated with DS.

Any differences between our results and those of AD GWAS meta-analyses (e.g., Hollingworth et al., 2011; Naj et al., 2011) are most likely due to the much lower number of samples used in our study. Because of this small sample size, we have not currently applied a multiple testing correction to our data, which we acknowledge to be a limitation of this study. AD in DS is still a relatively understudied area in the field of neurodegeneration, and this study highlights the need for larger collaborative studies that would improve our chances of dissecting the key genetic pathways which accelerate the onset of dementia. As studies in this field continue, we can identify further similar genetic risk factors between AD in the general population and DS. These similarities can guide us towards therapies which may benefit both populations.

Given the high occurrence of AD in people with DS, we have the opportunity to examine factors that affect AAO and disease progression, but only when large international collaborations are in place will we be able to fully take advantage of this opportunity. For this reason, we have made all of the genome-wide data available as PLINK files (Data S1), which we hope other researchers in this area will make use of in meta-analysis studies.

## Disclosure statement

Julie Williams has research collaborations with Elly-Lilly and Population Genetics Technologies Ltd (Cambridge) and has presented papers at conferences funded by Esai.

## Appendix A Supplementary data

Table S1 Analysed SNPs. SNPs were selected from previous GWAS meta-analyses (Hollingworth et al., 2011; Naj et al., 2011) and successfully genotyped by HumanOmniExpress-12v1_H in this study.

Supplementary Methods S1 Risk score analysis of SNPs from AD GWAS.

Table S2 Calculation of composite scores for risk analysis.

Table S3 Results for APOE, CLU, PICALM, BIN1, CR1, ABCA7, CD2AP, EPHA1, CD33 and MS4.

Table S4 Risk analysis comparison of AAO between upper and lower quintiles.

Data S1_1 PLINK data.

Data S1_2 PLINK data.

Data S1_3 PLINK data.
