# Supplementary material for: Evidence that PICALM affects age at onset of Alzheimer's dementia in Down syndrome
Source: Neurobiol Aging. 2013 Oct;34(10):2441.e1–5. doi: 10.1016/j.neurobiolaging.2013.03.018 (PMC3898582; doi:10.1016/j.neurobiolaging.2013.03.018)
Supplement: Supplementary Methods S1 — Risk score analysis of SNPs from AD GWAS. [file mmc2.docx]

Supplementary method.

10 genes were reported by Bettens et al. (2013) and Alzgene.org. They are CR1, BIN1, CD2AP, EPHA1, CLU, MS4A4A (MS4A6E), PICALM, ABCA7, APOE, and CD33. Except CR1, CD2AP and APOE, the SNPs reported were available in our data set. They did not report APOE SNPs. We located a common SNP rs405509 in our database that was found in both Hollingworth et al., (2011) and Naj et al., (2011) metaanalyses. We searched in the Broad Institute SNP Annotation and Proxy Search (http://www.broadinstitute.org/mpg/snap/) to find SNPs that were best linked with rs2274566 of CR1 and rs9296559 of CD2AP. rs9349407 is linked with rs9296559 and has r2 and D’ both equal to 1. No satisfactory linked SNP was found for rs2274566. We dropped the CR1 SNP from calculation of the composite score.

The OR for each SNP was obtained from Bettens et al. (2013). For every allele with the risk allele, we gave a score of 1 x OR; for 2 alleles 2 x OR was used; and zero for non-risk allele. For example, 2 A alleles in APOE rs405509 has a score of 2 x 1.45 = 2.9. We then screened the 67 cases with the genotypes of the 9 SNPs and calculated a composite score. (Table S2).

We use this score to correlate the age of onset using linear regression. In addition we compared the age of onset of the highest and lowest 20% of the score using Mann-Whitney U-test (Wilcoxon rank sum test) in R.

We did not attempt to calculate an OR between the highest and lowest quartile as carried out by Nalls et al. (2011) and Ripatti et al. (2011) as we did not have controls in this dataset. Of our 129 original samples, some cases did not have dementia. This is most likely because they have not reached the age of onset and given time, they would develop Alzheimer’s-type dementia. The only theoretical controls are DS cases who do not develop dementia beyond the mean dementia onset age.
